# Supplementary material for: Emergence and control of photonic band structure in stacked OLED microcavities
Source: Nat Commun. 2021 Oct 20;12:6111. doi: 10.1038/s41467-021-26440-3 (PMC8528838; doi:10.1038/s41467-021-26440-3)
Supplement: Supplementary file 4 — Supplementary Data 1 [file 41467_2021_26440_MOESM4_ESM.zip › OLED Simulation v2-1/OLED Simulation/Materials Data/Materials Database/rii-database-2019-02-11.zip!/database/info/main/H2O.html]

# Water and ice, H_2_O

Water is the most abundant compound on Earth's surface.

## External links

- Water - Wikipedia
- Ice - Wikipedia
- Properties of water - Wikipedia
- Optical properties of water and ice - Wikipedia
- Ice I_h_ - Wikipedia
- Water - NIST Chemistry WebBook
